# Efficacy of 4-dimensional hysterosalpingo-contrast sonography and X-ray hysterosalpingography in infertility

**DOI:** 10.12669/pjms.41.1.10743

**Published:** 2025-01

**Authors:** Meili Wu, Xue Gu, Juan Hao, Ruichao Miao

**Affiliations:** 1 Meili Wu Department of Gynecological Center, Qingdao Women and Children’s Hospital, Qingdao, Shandong Province 266000, P.R. China; 2 Xue Gu Department of Gynecological Center, Department of Obstetrics, Qingdao Women and Children’s Hospital, Qingdao, Shandong Province 266000, P.R. China; 3 Juan Hao Department of Gynecological Center, Qingdao Women and Children’s Hospital, Qingdao, Shandong Province 266000, P.R. China; 4 Ruichao Miao Department of Reproductive Center, Qingdao Women and Children’s Hospital, Qingdao, Shandong Province 266000, P.R. China

**Keywords:** 4-dimensional hysterosalpingo-contrast sonography, X-ray hysterosalpingography, Laparoscopy and dye test, Infertility, Fallopian tube

## Abstract

**Objective:**

To assess and compare efficacy of 4-dimensional hysterosalpingo-contrast sonography (4D-HyCoSy) and X-ray hysterosalpingography (HSG) for fallopian tube examination.

**Methods:**

Clinical data of patients with suspected tubal infertility, who underwent examinations in Qingdao Women and Children’s Hospital from September 2021 to December 2023, were retrospectively analyzed. Of them, 40 patients received laparoscopy and dye test+ 4D-HyCoSy (4D-HyCoSy group), and 36 patients received laparoscopy and dye test +HSG (HSG group).

**Results:**

The study included clinical data of 76 patients with a total of 149 fallopian tubes. Using laparoscopy and dye test observations as the gold standard, 4D-HyCoSy and X-HSG examinations identified 39 blocked tubes, 69 tubes with partial patency, and 41 tubes with patency. Compared with laparoscopy and dye test, the diagnostic concordance rate of 4D-HyCoSy was 70.51% (55/78), Kappa=0.546 (*P*<0.05); and the diagnostic concordance rate of HSG was 69.01% (49/71), Kappa=0.516 (*P*<0.05). Sensitivity, specificity, and accuracy of the diagnosis of obstruction, partial patency, and patency were comparable in the two groups (*P*>0.05). The incidence of pain and adverse reactions were significantly lower in the 4D-HyCoSy group compared to the HSG group (*P*<0.05).

**Conclusions:**

While both 4D-HyCoSy and HSG have high efficacy in fallopian tube examination, and are similarly accurate in evaluating tubal blockage, 4D-HyCoSy is associated with lower incidence of pain, and reduced rate of adverse reactions.

## INTRODUCTION

According to the World Health Organization (WHO), one n six people worldwide affected by infertility, which has a profound negative impact on physical and mental well-being of patients and their families.^1^,^2^ Studies show that the most common causes of infertility include ovulatory dysfunction, uterine abnormalities, and tubal disease.^3^,^4^ Laparoscopy with dye test is considered a gold standard for the diagnosis of infertility, as it can clarify the nature and severity of pelvic organ lesions, and manage pelvic adhesions.^5^ However, this is relatively invasive and costly procedure, which limits its clinical application.^5^,^6^

X-ray hysterosalpingography (HSG) is an important diagnostic measure for infertility that evaluates the shape of the uterus and can determine the degree of patency of the fallopian tubes.^7^ However, due to the use of X-ray radiation, HSG may potentially have certain adverse effects. Additionally, this procedure is not suitable for individuals who are allergic to iodine.^7^,^8^ With the development of ultrasound technology, 4-dimensional hysterosalpingo-contrast sonography (4D-HyCoSy) which is performed under ultrasound guidance gained popularity in clinical practice. This method can generate static images of the fallopian tubes in all directions, providing information on the location of blocked fallopian tubes and accurately evaluate their condition.^9^,^10^

Several studies have attempted to compare the efficacy of X-ray HSG and 4D-HyCoSy, with conflicting results. While some studies have reported that 4D-HyCoSy is associated with superior safety and accuracy for the detection of female upper genital tract pathologies,^11^,^12^ others found that both techniques were comparable in efficacy.^13^ This study retrospectively analyzed clinical data of patients with suspected tubal infertility who underwent laparoscopy with dye test in combination with either 4D-HyCoSy or X-ray HSG examination to clarify and compare efficacy of both techniques for the diagnosis of tubal infertility.

## METHODS

Clinical data from 76 suspected tubal infertility patients who underwent examinations in Qingdao Women and Children’s Hospital from September 2021 to December 2023 were retrospectively selected. Laparoscopy with dye test was used as the gold standard and all patients underwent laparoscopy with dye test. Patients who also underwent 4D-HyCoSy examination comprised the 4D-HyCoSy group, while patients who underwent X-ray HSG were included in the (HSG group).

### Ethical Approval:

This retrospective study was approved by the hospital ethics committee on Mach 29^th^ 2024, No. QFELL-YJ-2024-53.

### Inclusion criteria:

- Patient has normal sexual activity without taking contraceptive measures, has not been pregnant for more than one year, and meets the diagnostic criteria for infertility.^14^
- Patients who have been clean for three to seven days after menstruation without vaginal bleeding and have not had sexual intercourse for three days.
- Patient who underwent complete laparoscopy and dye test, and in addition, 4D-HyCoSy or HSG examination.
- Complete clinical data and clear contrast images.
- Age range from 20 to 45 years old.

### Exclusion criteria:

- Infertility due to the male factor.
- Patients with congenital uterine malformations such as uterine septum and unicorn uterus.
- Patients with a history of trauma to uterus and/or fallopian tubes.
**- Patients with uterine adhesions, pelvic adhesions, or uterine tumors.**
**- Patients with vaginal bleeding or pelvic infection.**
- Patients with congenital occlusion of the fallopian tubes.

### Diagnostic procedures:

All procedures were performed in a similar manner by the same group of doctors in a single centre.

*(1) 4D-HyCoSy:* Voluson S8 color ultrasound diagnostic instrument (GE Healthcare, Milwaukee, WI, USA) was used for the examination.^10^ Perfluoropropane human albumin microspheres (Hunan Ruikang Pharmaceutical Co., Ltd.) were selected as the contrast agent. Perfluoropropane human serum albumin microspheres (3ml) were diluted in physiological saline at 37°C for 20 ml before use. Patient was instructed to empty the bladder before the examination and lie on the operating table. Routine scanning of the uterus and adnexa, pelvic basic condition, and pelvic activity of the uterus and adnexa were done using a vaginal volume probe to determine the position of both ovaries. Routine intubation, injecting a water sac (1-1.5 ml) into the uterine cavity, and adjusting the size of the water sac through ultrasound monitoring were performed.

In cases of anxiety, patients were calmed down in a timely manner to avoid tubal spasms. 3D pre-scan was done first to determine whether the bilateral ovaries can be completely visualized and whether uterine cavity imaging can determine the presence of uterine malformations. Then, imaging parameters and four-dimensional mode were activated. Diluted contrast agent was injected at a uniform speed and the flow path of the contrast agent was dynamically observed. Dynamic images were stored, and 3D static images were captured. For suspected cases of blocked fallopian tubes, an appropriate amount of agent was injected under two-dimensional ultrasound monitoring. In case of resistance, appropriate pressure was applied, and the tube was repeatedly rinsed to unblock. In cases of muscle layer or parametrial reflux that affected the observation, patient was allowed to rest and the procedure was repeated. Adhesion status around the ovaries and pelvic cavity were explored in a comprehensive two-dimensional mode. After completing the procedure, physiological saline was injected into the uterine cavity.

*(2) X-ray HSG:* Patient was instructed to lie on the operating table and the balloon was placed. Slowly inject 8-15ml iohexol (GE Healthcare, Milwaukee, WI, USA) into the uterine cavity through X-ray fluoroscopy assisted catheterization, and observe the flow of iohexol in the uterine cavity and fallopian tubes.^15^ When the uterus and the fimbria of the fallopian tube were fully visualized, the best observation image was selected and the film was taken.

*(3) Laparoscopy and dye test:* Laparoscopic and dye test was performed under general anesthesia.^16^ After placing the laparoscope, the pelvic cavity was explored for pelvic adhesions, and 20 ml dose of methylene blue (Jumpcan, Taixing City, China) was injected into the uterine cavity through a dual-lumen hysterosalpingography catheter to observe tubal patency.

*Diagnostic criteria:*

For 4D-HyCoSy examination, fallopian tube was considered patent if there was minimal or no resistance during the contrast agent injection; contrast agent quickly filled the fallopian tubes and diffused into the pelvic cavity, and evenly distributed around the periphery of the ovary and intestinal space; the fallopian tube wall was smooth. Fallopian tube was considered obstructed if there was a significant resistance during injection, obvious pain; fallopian tubes were completely or partially not visualized; there was no contrast agent overflow at the fimbriae end. Partial patency was diagnosed if there was significant resistance, but contrast agent was able to pass through the fallopian tube if the pressure was increased; fallopian tubes had uneven thickness, and unsmooth tube walls; there was evidence of local twisting and coiling, stiff walking or angled lifting; a small amount of contrast agent was overflowing from the umbrella end, and a small amount of contrast agent was present between the ovaries and intestines.

For HSG examination, fallopian tube was considered patent if the contrast agent was flowing normally and the tube was clearly visible throughout the process. If the fallopian tubes were filled, and clearly visualized, and a small amount of contrast agent was discharged from the distal end, with some of the contrast agent entering the abdominal cavity, but the diffusion effect was not good, a diagnosis of partial patency was made. If the fallopian tube was filled without contrast agent development or if there was no diffusion in the abdominal cavity, the diagnosis of obstruction was made.

For laparoscopy and dye test procedure: Patency- no resistance when injecting methylene blue solution; no dilation of the fallopian tubes; methylene blue naturally flowed out from the fimbriae of the fallopian tube. Partial patency- some resistance during the injection of methylene blue solution, the fallopian tubes showed slight swelling and blue staining; a small amount of methylene blue flowed out of the fimbriae of the fallopian tube. Obstruction- substantial resistance when injecting methylene blue, with reflux; dilation and blue staining in the vaginal area; no flow of methylene blue from the fimbriae of the fallopian tube.

### Pain assessment criteria:

Pain level was evaluated using the Visual Analog Scale (VAS).^17^ A score of 0 points was considered as no pain; 1-3 points as mild pain; 4-6 points as moderate pain. A score of 7-10 indicated severe pain.

### Adverse reactions:

Incidences of nausea and vomiting, rash, pelvic inflammatory disease, and vaginal bleeding were recorded.

### Statistical Analysis:

SPSS version 22.0 (IBM Corp, Armonk, NY, USA) was used for analysis. Quantitative data were expressed as mean ± standard deviation, and independent sample t-tests was used for the analysis between the groups. Count data were expressed in the form of the number of use cases (%), and chi square test was used between the groups. The consistency test adopted Kappa test. *P*<0.05 indicated statistical significance.

The diagnostic value of tubal infertility was determined as follows: Sensitivity=true positive/ (number of true positive+number of false negative) × 100%; Specificity=true negative/ (number of true negative+number of false positive) × 100%; Accuracy= (true positive+true negative)/ (true positive+true negative+false positive+false negative) x 100%.

## RESULTS

Clinical data of 76 patients were retrospectively reviewed. Patients aged 20-41 years, with a mean age of 30.21 ± 5.41 years. Three patients had one fallopian tube removed due to ectopic pregnancy, thus a total of 149 fallopian tubes included in the analysis. Laparoscopy and dye test results was used as the gold standard. Based on the outcomes of laparoscopic assessment, 39 tubes were diagnosed as obstructed, 69 as having partial patency, and 41 as patent. A total of 40 patients (78 fallopian tubes) underwent 4D-HyCoSy examination (4D-HyCoSy group), and 36 patients (71 fallopian tubes) underwent X-ray HSG (HSG group). There was no statistically significant difference in the baseline data between the two groups (*P*>0.05) (Table-I).

**Table-I:** Comparison of baseline data between two groups.

Baseline data	4D-HyCoSy group (n=40)	HSG group (n=36)	t/χ^2^	P
Age (year)	30.85±5.12	29.50±5.69	1.088	0.280
Infertility years (years)	4.05±1.71	4.17±192	-0.280	0.780
Infertility type				
Primary infertility	11 (27.50)	12 (33.33)	0.306	0.580
Secondary infertility	29 (72.50)	24 (66.67)		
BMI (kg/m^2^)	23.02±2.87	22.83±2.20	0.315	0.754
History of childbirth				
Yes	27 (67.50)	26 (72.22)	0.200	0.655
No	13 (32.50)	10 (27.78)		
Marital status				
Married	34 (85.00)	29 (80.56)	0.264	0.607
Unmarried	6 (15.00)	7 (19.44)		
Cleanliness of vaginal discharge				
I	19 (47.50)	19 (52.78)	0.211	0.646
II	21 (52.50)	17 (47.22)		

Compared with laparoscopy and dye test, the diagnostic concordance rate of 4D-HyCoSy was 70.51% (55/78), Kappa=0.546 (*P*<0.05) (Table-II); and the diagnostic concordance rate of HSG was 69.01% (49/71), Kappa=0.516 (*P*<0.05) (Table-III). There was no significant difference in the sensitivity, specificity, and accuracy of the diagnosis of obstruction, partial patency, and patency between the two groups (*P*>0.05) (Table-IV).

**Table-II:** 4D-HyCoSy examination of fallopian tubes.

4D-HyCoSy	Laparoscopy and dye test			Total number of tubes

	Obstruction	Partial patency	Patency	
Obstruction	18	4	3	25
Partial patency	3	25	5	33
patency	2	6	12	20
Total	23	35	20	78

**Table-III:** HSG examination of fallopian tubes.

HSG	Laparoscopy and dye test			Total number of tubes

	Obstruction	Partial patency	Patency	
Obstruction	12	3	3	18
Partial patency	3	24	5	32
patency	1	7	13	21
Total	16	34	21	71

**Table-IV:** Sensitivity, specificity, and accuracy of 4D-HyCoSy and HSG examinations.

Methods	Obstruction			Partial patency			Patency		

	Sensitivity	Specificity	Accuracy	Sensitivity	Specificity	Accuracy	Sensitivity	Specificity	Accuracy
4D-HyCoSy	78.26% (18/23)	67.27% (37/55)	70.51% (55/78)	71.43% (25/35)	69.77% (30/43)	70.51% (55/78)	60.00% (12/20)	74.14% (43/58)	70.51% (55/78)
HSG	75.00% (12/16)	67.27% (37/55)	69.01% (49/71)	70.59% (24/34)	67.57% (25/37)	69.01% (49/71)	61.90% (13/21)	72.00% (36/50)	69.01% (49/71)
*χ^2^*	0.022	0.000	0.040	0.006	0.045	0.040	0.016	0.063	0.040
*P*	0.882	1.000	0.842	0.939	0.832	0.842	0.901	0.803	0.840

The incidence of pain in the 4D-HyCoSy group (50.00%) was significantly lower than that in the HSG group (72.22%) (*P*<0.05) (Table-V). 4D-HyCoSy examination was associated with significantly lower incident of adverse reactions (17.50%) compared to the HSG group (38.89%) (*P*<0.05) (Table-VI).

**Table-V:** Pain incidence rates.

Groups	Number of patients	Mild pain	Moderate pain	Severe pain	Overall incidence rate
4D-HyCoSy group	40	15 (37.50)	4 (10.00)	1 (2.50)	20 (50.00)
HSG group	36	15 (41.67)	7 (19.44)	4 (11.11)	26 (72.22)
*χ^2^*					3.916
*P*					0.048

**Table-VI:** Incidence of adverse reactions.

Groups	Number of patients	Nausea and vomiting	Rash	Pelvic infection	Vaginal bleeding	Overall incidence rate
4D-HyCoSy group	40	7 (17.50)	0 (0.00)	0 (0.00)	0 (0.00)	7 (17.50)
HSG group	36	11 (30.56)	1 (2.78)	1 (2.78)	1 (2.78)	14 (38.89)
*χ^2^*						4.335
*P*						0.037

## DISCUSSION

The current study showed that both 4D-HyCoSy and X-ray HSG can effectively identify obstruction of fallopian tubes with comparable accuracy (70.51% and 69.01%, respectively). There was no significant difference in the diagnostic sensitivity, specificity, and accuracy between the two examination methods for patency, obstruction, and partial patency of the fallopian tubes. The results are same as those of Wang et al^13^ and Yang et al.^18^ However, we also found that 4D-HyCoSy examination was associated with significantly lower incidence of pain and adverse reactions compared to X-ray HSG.^7^-^10^,^18^,^19^

HSG is currently a commonly used diagnostic method for tubal infertility in clinical practice.^7^,^8^,^19^ This technique allows to observe the distribution of contrast agent in the uterus and fallopian tubes by injecting oil contrast agent to detect obstruction.^8^,^19^ However, HSG examination is prone to interference from intestinal gas during the imaging process, and the observation plane is limited, making it impossible to assess the shape of fallopian tubes from various angles and sections.^18^,^19^ 4D-HyCoSy is a real-time 3D imaging technique that can display the entire process of contrast agent entering the uterine cavity through the catheter and progressing along the fallopian tubes in real time. 4D-HyCoSy provides clear and realistic imaging that is dynamic and intuitive. Additionally, storing images allows for multi-plane and multi-angle post-examination processing to avoid interference from retrograde flow in the parametrial and muscular layers.^20^-^22^

A retrospective study by Gao YB et al.^20^ that compared data of 195 patients (385 fallopian tubes) who underwent TVS 4D-HyCoSy and 72 patients (144 fallopian tubes) who underwent laparoscopic examination, showed that 4D-HyCoSy had high sensitivity, specificity, positive predictive value, negative predictive value, and Youden index (97.7%, 86.7%, 98.4%, 81.3%, and 0.84, respectively). He et al.^21^ also demonstrated that compared to laparoscopy and dye test as a reference standard, 4D-HyCoSy demonstrated high performance in diagnosing tubal patency, with sensitivity, specificity, positive predictive value, and negative predictive value of 88.4%, 85.2%, 90.5%, and 82.1%, respectively. Wang et al.^23^ confirmed that the overall consistency between 4D-HyCoSy and laparoscopic staining catheterization was 92.9%, while the sensitivity and specificity of 4D-HyCoSy using laparoscopic staining catheterization as the reference standard were 93.8% and 92.2%, respectively. Together, these reports further confirm high sensitivity, specificity, and accuracy of 4D-HyCoSy in the diagnosis of tubal infertility, which is consistent with the results of our study.

Li et al has shown that more than 90% patients undergoing 4D-HyCoSy felt pain, but only 0.5% patients felt severe pelvic pain that was relieved within 30 minutes.^24^ Similarly, we further confirmed that 4D-HyCoSy was less painful and had a lower adverse reaction rate compared with X-ray HSG. HSG examination exposes patients to X-rays. Long-term repeated hysterosalpingography under X-rays is harmful and may cause a series of complications. 4D-HyCoSy avoids iodine oil allergy and radiation that may be caused by X-ray HSG. In addition, studies have shown that 4D-HyCoSy is superior to HSG in its ability to evaluate fallopian tube patency and does not impact subsequent conception time.^22^,^25^ Moreover, pregnancy can often be achieved in the next physiological cycle, since for some patients, the procedure can restore the patency of fallopian tubes that are blocked due to tubal adhesions.^26^,^27^ 4D-HyCoSy has the advantages of safety, no need for radiation, and is not limited by angle, direction, or space during the imaging. Multiple angles can be used to observe the structure, course, patency, and obstructed area of the fallopian tubes, and makes it possible to recanalize the tubes.^26^-^28^ However, it is important to note that current study did not analyze the impact of 4D-HyCoSy on the pregnancy status of infertile patients. Further research is needed to verify this potential effect of 4D-HyCoSy. Overall, 4D-HyCoSy is a dynamic, real-time, multidimensional diagnostic method for evaluating tubal patency. The results of this study provide more evidence support for the clinical application of 4D-HyCoSy, as it is as efficient as X-ray HSG but with a lower rate of pain and adverse reaction.

### Limitation:

Firstly, this is a single center retrospective analysis with a small sample size, which may have resulted in selection bias. Secondly, 4D-HyCoSy and HSG images may be influenced by human or technical factors. Thirdly, the observed research indicators in this study only focus on the patency and obstruction site of the fallopian tubes, without conducting compliance statistics and comparisons on indicators such as fallopian tube effusion, pelvic adhesion, and uterine cavity lesions.

## CONCLUSION

Both 4D-HyCoSy and X-ray HSG have high efficacy in the diagnosis of infertility, with comparable accuracy in evaluating tubal blockage. At the same time, 4D-HyCoSy examination is associated with less pain and lower incidence of adverse reactions.

### Authors’ contributions:

**MW:** Conceived and designed the study, literature search, prepared the manuscript.

**XG**, **JH** and **RM:** Collected the data, performed the analysis and did Review.

All authors have read and approved the final manuscript and are responsible for the integrity of the study.
